# Come on Baby, Light My Fire: Sparking Further Research in Socio-Affective Mechanisms of Music Using Computational Advancements

**DOI:** 10.3389/fpsyg.2020.557162

**Published:** 2020-12-08

**Authors:** Ilana Harris, Mats B. Küssner

**Affiliations:** ^1^Centre for Music and Science, Faculty of Music, University of Cambridge, Cambridge, United Kingdom; ^2^Department of Musicology and Media Studies, Humboldt-Universität zu Berlin, Berlin, Germany

**Keywords:** socio-affective behavior, computational methods, joint music-making, prosocial behavior, emotional contagion

## Musical Engagement: Socio-affective Underpinnings

Socio-affective behavior is entangled in our experience of music (Devroop, 2012; Koelsch, 2014; Aucouturier and Canonne, 2017; Saarikallio, 2019). Joint musical engagement, or making and listening to music with others, was found to result in increased prosocial tendencies (Kirschner and Tomasello, 2010; Rabinowitch et al., 2013; Cirelli et al., 2014) and is thought to occur due to overlapping mechanisms underpinning interactive musical behavior and empathically driven prosocial behaviors (Rabinowitch et al., 2012; Clarke et al., 2015; Saarikallio, 2019). In this paper, we present opportunities for experimental investigation of emotional contagion, a specific subprocess hypothesized to lie at this overlap, and highlight ways to improve understanding of *how* joint musical engagement may promote prosocial behaviors.

Socio-affective components of joint musical engagement have been postulated following empirical investigation of joint music-making and group music-listening (Egermann et al., 2011; Rabinowitch et al., 2013) and hinge on subprocesses including affective alignment, where joint expression of emotion among interlocutors allows for facilitated transfer of semantic and affective content (Cross, 2005; Bharucha et al., 2012; Rabinowitch et al., 2012; Vesper et al., 2017). In this sense, affective alignment may contribute to higher-level processes of musical interaction such as shared intentionality by ensuring that members are working toward a common musical goal in real time and have “coordinated action roles for pursuing that shared goal” (Tomasello et al., 2005, p. 680) through upregulating constituent socio-affective behaviors (e.g., other-directed behaviors) that help individuals ascertain their interlocutor's internal state and align their behavior accordingly (Cross et al., 2012). Joint music-making's positive influence on socio-affective behaviors in non-musical contexts suggests that psychosocial processes underpinning musical interaction may overlap with those involved in non-musical interaction, and that co-activation of these overlapping structures may result in prosocial transfer effects (Kirschner and Tomasello, 2010; Cross et al., 2012; Saarikallio, 2019).

Scientific inquiry probing the effects of musical engagement on prosociality has risen in prevalence in recent years; particularly, musical engagement's influence on prosocial behaviors underscored by empathy has gained considerable traction in music psychology and related fields (King and Waddington, 2017; Davis, 2018; Riess et al., 2018). Empathy may be defined as “the ability to produce emotional and experiential responses to the situations of others that approximate their responses and experiences” (Rabinowitch et al., 2013, p. 485) and is a core component of social cognition comprising both slow (controlled) and fast (automatic) psychological subprocesses (i.e., dual process theory; Lieberman, 2007; Batson, 2009) that ultimately “constitute a causal force in motivating prosociality towards other conspecifics” (Decety et al., 2016, p. 371). Slow processes are “evaluative,” requiring top-down cognitive assessment, while fast processes are immediate, “automatic detection” of social signals; separate neural representations for fast and slow processing of social information have been proposed accordingly (i.e., “mirror neuron system” and “mentalizing system”; Vogeley, 2017). Emotional contagion, a subprocess of empathy, is defined as automatic mimicry of another's behavioral cues associated with a particular affective state; it is thought to foster survival through increasing recognition of and successful communication between conspecifics, and underpins the capacity to build and maintain human attachment bonds (de Vignemont and Singer, 2006; Feldman, 2017; Prochazkova and Kret, 2017). Though theoretical study of emotional contagion in music has begun, there is a lack of experimental study that causally explains how automatic detection of socio-affective signals influences our experience of music (Miu and Vuoskoski, 2017). Investigation of emotional contagion during musical interaction is critical to understanding how relationships between co-performers may be similar to other types of social relationships (e.g., through attachment bonds) and, consequently, how joint musical engagement may lead to upregulated other-directed behaviors such as those that arise within a particular social relationship.

Experimental investigation of emotional contagion is practically difficult because it requires simultaneous measurement of interlocutors' complex emotion states in interactionist paradigms; this matter is further complicated in the context of music, where substantial ecological validity is needed to elicit behaviors of interest (e.g., empathy-promoting musical components; Rabinowitch et al., 2013). In the following two sections, we introduce research from related fields incorporating computational techniques for measuring behavioral and physiological correlates of emotional contagion; we situate such techniques in the context of music psychology and suggest avenues by which they may be incorporated into existing experimental paradigms to triangulate investigation of socio-affective processes using behavioral, physiological, and social signal processing, as has been done across numerous subfields of psychology (Pantic and Vinciarelli, 2015; Azevedo et al., 2016; Sutherland et al., 2017; de Barbaro, 2019; Oswald et al., 2020).

## The Frontier: Behavioral Cues

The following section outlines possibilities for investigating socio-affective components in music using computational methods for behavior recognition drawn from research in computer science and the behavioral sciences. First, facial cues are an important behavioral cue for emotional expression in music (Thompson et al., 2008, 2010; Livingstone et al., 2009; Waddell and Williamon, 2017). Approximately 95% of automatic emotion recognition literature relies on facial cues, which has led to applicability of these techniques to an expanding number of datasets (Noroozi et al., 2018). Computational emotion recognition using facial cues has been incorporated into the study of empathic behavior in group settings. For instance, Kumano et al. (2011, 2014, 2015) conducted a series of experiments to see if empathic interactions could be predicted based on facial data from video recordings of four-person meetings. Their Naive Bayes Network Model was able to predict empathy state given facial expression information across time and improved when parameters such as reaction time in mirrored expression between interlocutors and head gesture annotations were added. Scientific study of music has not yet incorporated computational determination of joint emotional expression epochs from facial cues; this is likely to be a fruitful area of inquiry, involving relay of complex affective information at the intersection of individually, socially, and musically driven systems.

Though literature examining communication of emotion through body as opposed to facial cues in non-musical contexts is lacking, several studies have found that determination of emotion state is modulated by body posture/movement (Aviezer et al., 2008a,b; Martinez et al., 2016). In musical settings, visual content, often in the form of body movement, plays a critical role in conveying affective information (Vines et al., 2011; Vuoskoski et al., 2014, 2016). Computational analysis of body position/gesture using motion capture experiments has incurred important findings with respect to joint emotional expression and audience-perceived emotion (Burger et al., 2013; Chang et al., 2019). Still, analyses of body postures/gestures across various cultural and developmental contexts and further determination of indices that convey socio-affective information are necessary to better understand their role in both musical interaction and potential transference to non-musical interaction.

Following research on prosocial behaviors as a consequence of joint music-making, similar outcomes of music listening have begun to be studied (Ruth and Schramm, 2020). Continuous self-report of emotion by audience members during live concert settings is a promising experimental tool (Egermann, 2019). These measures collect rating data simultaneously with and continuously throughout the stimuli's presentation and may be able to achieve the temporal specificity needed in order to determine instances of affective alignment between participants. Moreover, continuous measurement of self-reported affect supports various forms of rating interfaces, including linear potentiometers (Vines et al., 2011; Baytaş et al., 2016), binary trigger buttons (Baytaş et al., 2019), and four-quadrant valence-arousal joysticks (Sharma et al., 2020; or its digital analog in Egermann, 2019). Furthermore, such interfaces may be attached to the participant (i.e., wearables) such that implementation in a paradigm involving movement is possible. In addition, top-down cameras can provide useful visual displays of crowd behavior, as evinced in analyses of pedestrian movement (Xu et al., 2020); concerning non-coordinated movement of audiences, this line of research within computer science could nicely complement existing methods in motion tracking (e.g., analysis of head movement in Swarbrick et al., 2019).

## The Future: Emerging Data Sources and Analyses

Music serves a number of social functions in everyday listening (Sloboda and O'Neill, 2001). Recently, social surrogacy was added as a potential reason for musical listening, extrapolating online listener behavior to internal processes (Schäfer and Eerola, 2020). Greenberg and Rentfrow (2017) list a number of avenues by which social media and streaming data can be used within music psychology; implementing several of these analyses in tandem could be well-suited to studying socio-affective behavior. For example, combining analyses of song-specific emotion data from Spotify APIs, listeners' comments on social media and self-report data gathered from online surveys could help determine individual differences in socio-emotional components of music listening. In addition, experience sampling methods (ESMs) have become increasingly popular for administering repeated surveys of everyday musical experience (e.g., Juslin et al., 2008; de Barbaro, 2019), with more recent ESM interfaces allowing for user mobility and nuanced user input (e.g., Randall and Rickard, 2017). Housing state-of-the-art digital self-assessment scales for emotion within existing ESMs could help bolster outcome evaluations and uncover relationships between socio-affective components in everyday musical behaviors (Betella and Verschure, 2016; Juslin, 2016).

Detecting emotion from acoustic properties of music has been extensively researched; for instance, tempo and mode tend to be good indicators of perceived emotion (Eerola et al., 2013). However, computational methods for music emotion recognition have tended to favor certain features over others (e.g., timbre accounting for over 60%; Yang et al., 2019). Recently, researchers have begun to develop software packages for emotion recognition in music, which include fine-grained features such as specific textural shifts and articulations (Panda et al., 2018). Such software could provide important contextual affective information in existing joint music-making paradigms. In addition, natural language processing (NLP) of song lyrics is a burgeoning area of research in music psychology (Anglada-Tort et al., 2019). NLP could be useful for identifying empathic tendencies in group songwriting, a prevalent music therapy intervention (e.g., using language style synchrony as a proxy for empathy as in Lord et al., 2015).

Lastly, behavioral tasks that can robustly quantify socio-emotional components in various populations after engagement in social music activities are needed. Several studies to date have developed novel tasks or adapted tasks from other disciplines to achieve this end (Rabinowitch et al., 2013; Reddish et al., 2014; Brown, 2017). In social neuroscience, researchers have investigated mechanisms underlying evolutionarily advantageous socio-affective behavior through experimental paradigms targeting social modulation of threat response (DeVries et al., 2003; Coan et al., 2006). Automated stress recognition *via* analyses of multimodal physiological and motion data has begun to show potential for validated use in social science research (Hovsepian et al., 2015). Further, higher-order pattern detection of heart rate variability (HRV) has been used to predict interpersonal affective alignment at levels above chance (McCraty, 2017). In the near future, such methods could be incorporated into behavioral cooperation tasks following joint music-making paradigms in order to assess transfer effects on socio-affective processing in non-musical contexts.

## Concluding Thoughts

Several precautions should be taken when incorporating emotion detection techniques into scientific study of music. A general framework for ethical (e.g., intrinsic biases due to demographically limited training) and practical (e.g., overfitted algorithms) considerations for using computational techniques in social science research is covered in a review paper by Martinez (2019). Concerns specific to music psychology include the following. First, non-verbal displays of emotion in musical settings may present differently than in idealized non-musical settings (e.g., differing behavioral cues for real vs. acted emotions as in Wilting et al., 2006; overlapping basic emotions as in Juslin et al., 2011; Juslin, 2013; Akkermans et al., 2019); an affective taxonomy appropriate for the given research question should be carefully determined and cross-checked with each session of algorithmic fine-tuning. Furthermore, it is likely advantageous to limit stimuli to a particular genre or song in order to conserve behavioral cue utilization among both performers and listeners (Juslin, 2000) and de-escalate computational complexity (Lange and Frieler, 2018).

This article has summarized recent developments in music psychology and related fields that may be applied to detecting emotional contagion in music. We have discussed this research in terms of how it may be incorporated into existing experimental paradigms in scientific studies of music. We hope to encourage further findings regarding the means by which various forms of musical engagement can result in positive prosocial consequences for a broader population.

## Author Contributions

IH conducted literature reviews in order to gather necessary evidence and to generate initial drafts. MBK assisted argument development and expanded source material. Both authors approved the final version of the manuscript.

## Conflict of Interest

The authors declare that the research was conducted in the absence of any commercial or financial relationships that could be construed as a potential conflict of interest.
